# Combined dapagliflozin and pioglitazone therapy in diabetic nephropathy: no added benefit beyond monotherapy in inflammation and fibrosis

**DOI:** 10.1007/s00210-025-04613-x

**Published:** 2025-09-26

**Authors:** Cinakova A, Vavrincova-Yaghi D, Vavrinec P, Krenek P, Klimas J, Kralova E.

**Affiliations:** Comenius University Bratislava, Faculty of Pharmacy, Department of Pharmacology and Toxicology, Kalinciakova 8, SK-832 32 Bratislava, Slovakia

**Keywords:** Diabetic nephropathy, Inflammation, Fibrosis, Dapagliflozin, Pioglitazone, Rat

## Abstract

Diabetic nephropathy (DN) is currently a leading cause of end-stage renal disease. Both dapagliflozin and pioglitazone have shown protective effects on organ damage in diabetes, even beyond their blood glucose-lowering properties. This study aimed to assess whether the simultaneous activation of PPARγ and inhibition of SGLT2 cotransporters provide additive protection against inflammation and fibrosis which are highly engaged in the progression of DN in experimental type 1 diabetes mellitus. Diabetes was induced in Wistar rats using streptozotocin (55 mg/kg, i.p.), and the animals received daily chow containing dapagliflozin (10 mg/kg), pioglitazone (12 mg/kg), or their combination. Six weeks after streptozotocin administration, molecular, histological and immunohistochemical analyses were performed in the excised kidneys. In the kidneys of diabetic rats, disruption of renal function markers was accompanied by increased macrophage infiltration and collagen deposition. Both pioglitazone and dapagliflozin decreased proinflammatory markers expression (IL1b, IL6, Cox2, Tnfα) and CD68-positive areas. Both monotherapies positively modulated Tgfβ, HGF and Agtr1 expression, leading to fibrosis reduction and morphological normalisation. Similarly to pioglitazone, the combined therapy reduced α-SMA-positive areas. Despite an additive antihypertensive effect, the combination maximally reached the effect of the monotherapies in other measured parameters. Overall, both dapagliflozin and pioglitazone are renoprotective in monotherapy, but their combination does not provide additional benefits in reducing inflammation, fibrosis or restoring kidney function.

**Supplementary Information:**

The online version contains supplementary material available at 10.1007/s00210-025-04613-x.

## Introduction

Long-term diabetes is associated with severe microvascular complications, including diabetic nephropathy (DN), which remains the leading cause of end-stage renal disease (ESRD) worldwide despite significant advances in diagnostics and treatment (Tahara and Takasu 2018; Varghese and Jialal 2023). The early stages of DN are characterised by glomerular and tubular hypertrophy, along with hyperfiltration, which eventually progresses to micro- and macroalbuminuria, accompanied by a decline in the glomerular filtration rate (Jain 2012). The pathogenesis of DN is multifactorial, where chronic hyperglycaemia drives both metabolic and hemodynamic abnormalities (Zabad et al. 2019). Progressive kidney diseases, including DN, typically culminate in kidney fibrosis, characterised by extensive destruction of renal tissue and functional deterioration. A central mediator of this process is transforming growth factor-β (TGF-β), which plays a pivotal role in the development of DN-related glomerulosclerosis and interstitial fibrosis (Braga Gomes et al. 2014). Additionally, chronic low-grade inflammation in diabetes, driven by inflammatory cells and cytokines, significantly contributes to the activation of key pro-fibrotic pathways (Kanasaki et al. 2013). 

Sodium-glucose cotransporter 2 (SGLT2) inhibitors are glucose-lowering agents that exhibit renoprotective effects through both glucose-dependent and independent mechanisms (Kawanami et al. 2017). Dapagliflozin, the first approved SGLT2 inhibitor, significantly reduces albuminuria and serum creatinine levels, thereby decreasing the need for kidney replacement therapy by over 40%. The underlying molecular mechanisms responsible for these protective effects may involve hemodynamic modulation, as well as attenuation of oxidative stress and inflammatory pathways (Klen and Dolžan 2023). SGLT2 inhibitors have been shown to modulate inflammasome activity, leading to decreased cytokine production in macrophages (Kim et al. 2020). By reducing inflammatory cell infiltration, selective SGLT2 inhibition mitigates the development of renal interstitial fibrosis independently of glycaemic control, underscoring the critical role of its antifibrotic properties (Castoldi et al. 2020).

Besides the insulin sensitising and regulatory functions in metabolism, peroxisome proliferator–activated receptor gamma (PPARγ) are key modulators of both innate and adaptive immune responses, as they are expressed in different types of immune cells, including macrophages (Straus and Glass 2007). However, chronic hyperglycaemic conditions reduce the expression levels of PPARγ in the glomeruli (Zheng et al. 2002). Indeed, PPARγ deficient mouse macrophages show an increase in cytokines production such as TNF-α, IL1-β, IL-6 and IL-12 (Heming et al. 2018). PPARγ agonists suppress the polarisation of immunoreactive macrophage polarisation, which directly contributes to DN-related glomerulotubular injury, leading to fibrosis and proteinuria (Rayego-Mateos et al. 2020; Toobian et al. 2021). Numerous experimental and clinical studies have demonstrated the therapeutic potential of thiazolidinediones in preventing T2DM-related DN progression, though their use is limited by the risk of fluid retention (Papaetis 2022). Combining thiazolidinediones with dapagliflozin may offer a strategic advantage, as SGLT2 inhibitors promote sodium excretion, potentially mitigating peripheral oedema (Han et al. 2018). Notably, even subtherapeutic doses of pioglitazone and other thiazolidinediones, which do not fully normalise glycaemia, exert significant antifibrotic effects by counteracting inflammation and other pathological pathways involved in DN development (Toblli et al. 2011).

Slowing the decline in renal function has become a key focus in DN research, highlighting the urgent need for new strategies to prevent the progression of diabetic kidney disease (Kanasaki et al. 2013). It has been well-established that combining antidiabetic agents with different mechanisms of action represents the most effective treatment strategy (Nathan et al. 2009). Given the potential of dapagliflozin and pioglitazone to mitigate inflammatory and fibrotic pathways independently of their glucose-lowering effect, these agents may offer effective renal protection under hyperglycaemic conditions in T1DM. In this study, we hypothesised that simultaneous SGLT2 inhibition and PPAR-γ activation would additively alleviate inflammation and fibrosis in DN, thereby normalising renal function in early-stage experimental T1DM.

## Materials and methods

### Animals

The study was conducted using 3-month-old male Wistar rats (220–250 g) sourced from a breeding station in Dobrá Voda, Slovak Republic. Experimental procedures were performed following European animal research laws (European Communities Council Directive 2010/63/EU). The experimental protocol was approved by the Ethics Committee of the Faculty of Pharmacy, Comenius University, and the State Veterinary and Food Administration of the Slovak Republic (protocol No: Ro-1636/17–221, 28 April 2017). All procedures were carried out by certified personnel. The rats were given unrestricted access to chow and tap water. The experimental T1DM was induced by a single intraperitoneal injection of streptozotocin (STZ, 55 mg/kg, Sigma-Aldrich, St Louis, MO, USA) following overnight fasting. STZ was dissolved in a 0.1-mol/l citrate buffer (pH 4.5). After 72 h, diabetes was confirmed by measuring blood glucose from the tail using an Accutrend Plus glucometer (Roche, Switzerland). Animals with a fasting blood glucose concentration of 12 mmol/l were considered to be diabetic. The STZ-treated rats were randomly divided into four groups: rats without treatment (STZ, *n* = 10) and STZ rats with the administration of Dapa (Dapa, *n* = 12, 10 mg/kg), Pio (Pio, *n* = 12, 12 mg/kg) and their combination DapaPio (DapaPio, *n* = 12, Dapa 10 mg/kg, Pio 12 mg/kg) mixed in rat chow. Both the STZ-treated and control (*n* = 10) groups received standard chow. The experiment was conducted for 6 weeks. Before the day of termination, the rats were placed in individual metabolic cages for 24 h to measure water intake and urine output.

### Blood pressure measurement

Arterial blood pressure was assessed using the tail-cuff method in conscious animals pre-warmed to 37 °C, conducted 1 day prior to sacrifice. Measurements were obtained with a non-invasive blood pressure module (NIBP Controller, ADInstruments, Spechbach, Germany), connected to a manometer and a PowerLab 8/30 data acquisition system. For each data point, five recordings were analysed, and mean values were calculated. Data analysis was performed using Chart 5 software for Windows (ADInstruments, Spechbach, Germany).

### Sample collection

After 6 weeks, the rats were sacrificed by asphyxiation in a carbon dioxide chamber. The right kidney of each rat was fixed in 4% formaldehyde for histopathological and immunohistochemical analysis, while the left kidney was excised, frozen in liquid nitrogen, and stored at − 80 °C for further examination. Blood samples were collected from the abdominal aorta, centrifuged for 10 min at 4 °C after 1 h, and the serum was stored at − 80 °C for biochemical analysis. Urine samples were centrifuged for 15 min to remove particulates and stored at − 80 °C until further testing.

#### Assessment of biochemical parameters

Fasting blood glucose levels were measured from tail blood samples using an Accutrend Plus glucometer (Roche). Renal function was evaluated by assessing common parameters, including serum creatinine, blood urea nitrogen, and urinary proteins (Hatanaka et al. 2016). These analyses were performed by a certified diagnostic laboratory (synlab Slovakia, s.r.o., Slovak Republic). Creatinine clearance was calculated according to Kumari et al. (2021) and expressed in ml/min/kg of rat body weight.

### Real-time reverse transcription-polymerase chain reaction

Total RNA was extracted from left kidney tissue using Tri Reagent (Sigma, USA) and verified by agarose gel electrophoresis. RNA was then reverse-transcribed into cDNA using the High Capacity cDNA RT Kit with RNAse inhibitor (Applied Biosystems, USA). Real-time PCR was conducted using SYBR Green (SYBR Select Master Mix, Life Technologies, USA) on the StepOne Plus Real-Time PCR System (Life Technologies, USA), following the manufacturer’s instructions. Gene expression was assessed using gene-specific primers (Table 1). All primers were validated to produce a single PCR product with the expected amplicon length. β2-microglobulin (β2m) and Actb served as reference genes. Mean PCR efficiency and quantification cycles (Cq) were calculated using LinRegPCR software (version 2018.0), and efficiency-corrected relative abundance was calculated via the Pfaffl method (Pfaffl 2001).

**Table 1:** Primer sequences used in quantitative real-time PCR

Gene	GenBank PCR accession number	Primer sequence (5´−3´)	PCR product length (bp)
Actb	NM_031144.3	Forward: CCGCGAGTACAACCTTCTTG	81
		Reverse: GCAGCGATATCGTCATCCA	
Agtr1a	NM_030985.4	Forward: ATCTCGCCTTGGCTGACTTA	98
		Reverse: ACATAGGTGATTGCCGAAGG	
B2m	NM_012512.1	Forward: ATGGAGCTCTGAATCATCTGG	105
		Reverse: AGAAGATGGTGTGCTCATTGC	
Col1a1	NM_053304.1	Forward: AAGTCATAGGAGTCGAGGGAC	177
		Reverse: AGGACATCTGGGAAGCAAAGT	
Cox2	NM_011198.5	Forward: AGATCAGAAGCGAGGACCTGG	158
		Reverse: TGGGAGGATACACCTCTCCAC	
Hgf	NM_017017.2	Forward: TCAGCGCTGGGATCAGCAGACA	122
		Reverse: TGTAGCACCATGGCCTCGGCTT	
Il1b	NM_031512.2	Forward: AATCCCTGTGGCCTTGGG	96
		Reverse: GGATCCACACTCTCCAGCTGCAGG	
Il6	NM_012589.2	Forward: TCTCTCCGCAAGAGACTTCC	94
		Reverse: GTCTCCTCTCCGGACTTGTG	
Kim1	NM_001173393.3	Forward: AGGCCTCCTGCTGCTTCTTCCA	129
		Reverse: CGGCCCCAACATGTCGTTGTGA	
Nfkb	NM_001276711.1	Forward: TCCTTTCGGAACTGGGCAAA	111
		Reverse: AGGTATGGGCCATCTGTTGAC	
Nfkbia	NM_001105720.2	Forward: GAGGACGGAGACTCGTTCCT	150
		Reverse: GGTGATCACAGCCAAGTGGA	
Nphs1	NM_022628.1	Forward: TGCACCGTTGACGCCAATCCC	122
		Reverse: AGACGCCCCGTGGATCCCTT	
Tgfb	NM_021578.2	Forward: CCAAGGAGACGGAATACAGG	101
		Reverse: GTTTGGGACTGATCCCATTG	
Tnfa	NM_012675.3	Forward: AACTTCGGGGTGATCGGTCCCA	135
		Reverse: TACGACGTGGGCTACGGGCTT	

#### SDS‑PAGE and western blotting

Samples of left kidney tissue were rapidly frozen in liquid nitrogen and homogenised in a buffer containing 10 mM Tris–HCl (pH 7.4), 0.125 M sucrose, 1 mM EDTA-Na, 10% sodium dodecyl sulphate (SDS), and 1 mM phenylmethylsulphonyl fluoride. The samples were then subjected to SDS–polyacrylamide gel electrophoresis on a 12% gel and transferred to a polyvinylidene fluoride (PVDF) membrane (Immobilon PR, Millipore Corporation, USA). After blocking with 5% non-fat dried milk in TBST, the membranes were incubated with specific antibodies, including TNFα, IL1β and IL6 (ab9324, ab9787, ab6617; Abcam, Cambridge, UK). Actin beta (A2066; Sigma–Aldrich, St. Louis, USA) was used as the loading control. Immunoreactive proteins were detected using a chemiluminescent substrate (Immobilon Forte Western HRP substrate; Millipore Corporation, USA), visualised and quantified using UVITEC Imaging Systems (Uvitec Limited, Cambridge, UK). The resulting density values were normalised to the appropriate loading controls and calculated to the average control level to allow comparison of the protein amount between groups.

#### Histopathological analysis

Right kidney samples, embedded in paraffin blocks, were sectioned into 4-μm slices using a microtome (Leica) with Feather A35 razor blades (Bamed). The sections were deparaffinised in xylene, rehydrated through a graded ethanol series and stained with hematoxylin and eosin (H&E) and Picro Mallory trichrome (PMT). The sections were then dehydrated, rinsed in xylene and mounted with coverslips (Waldemar Knittel Glasbearbeitungs GmbH) using DPX mounting medium (Sigma-Aldrich). Sections were examined under a light microscope (Optika®) at magnifications ranging from 40 × to 400 ×. Morphological measurements were performed using the software Optika Proview, analysing 20 glomeruli from different non-interfering fields per sample, including the glomerular tuft, Bowman’s space, and capsule surface. Blue deposits of collagen were observed in the renal interstitial area. The percentage of collagen-positive areas was measured by selecting four non-interfering fields per each section (mean ± S.E.M of four fields). Analysis of renal images stained with Picro Mallory trichome was performed using ImageJ software (Image Processing and Analysis in Java).

### Immunohistochemistry analysis

For immunohistochemical (IHC) staining, we used 3-μm sections. Paraffin sections were dewaxed, hydrated and immersed in antigen retrieval (0.1 M Tris/HCl buffer, pH 9.0, at 80 °C). Blocking of endogenous peroxidase with 0.1% hydrogen peroxide was followed by incubation with anti-CD68 (cell signalling, 1:200 dilution) and anti-α-SMA (Dako, 1:200) primary antibodies at 4 °C overnight. This was followed by incubations with the appropriate secondary and tertiary antibodies (polyclonal rabbit anti-mouse/goat anti-rabbit/rabbit anti-goat immunoglobulins/HRP, Dako) at room temperature. Negative controls for immunohistochemistry included normal sera of the same species as the primary antibody. The immunoreactions were visualised with 3,3-diaminobenzidine (DAB, Dako), and finally counterstained with Harris hematoxylin, dehydrated, and mounted. A distinct brown-coloured reaction either within the cytoplasm or the nucleus was observed. CD68-positive cell numbers/α-SMA-positive areas were determined by randomly analysing 20 non-interfering fields per each section (magnification 400 ×).

#### Statistical analysis

Results are presented as the mean ± standard error of the mean (SEM). Data normality within groups was evaluated with the Shapiro–Wilk test. For normally distributed data, one-way ANOVA was performed, followed by the LSD (least significant difference) post hoc test. For non-normally distributed data, the Kruskal–Wallis test was used, with Dunn’s post hoc test applied as appropriate. The correlation analyses were conducted using Pearson’s or Spearman’s tests, depending on the distribution of the data. Statistical analyses were conducted using GraphPad Prism 10 and SPSS 16.0, with significance defined as *p* < 0.05.

## Results

### Biometric parameters

**Fig. 1:**
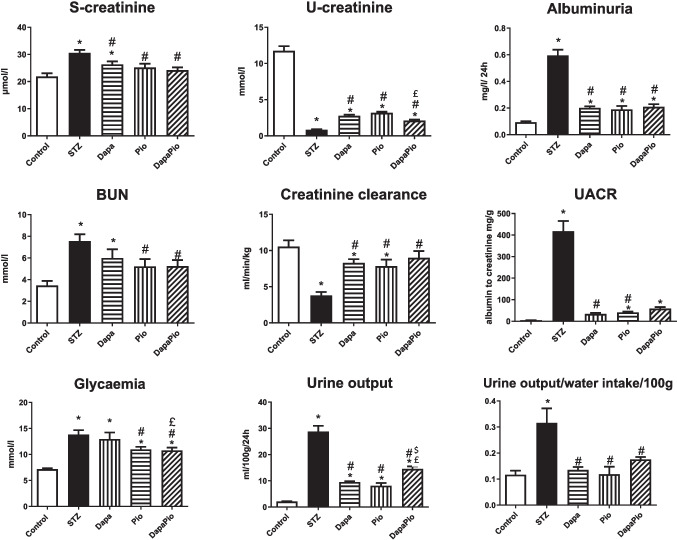
Effect of the therapy on biochemical parameters and markers of diabetic nephropathy. BUN—blood urea nitrogen; S-creatine—serum creatinine; U-creatinine—urine creatinine; UACR—urine albumin to creatinine ratio. Groups labelling: STZ—streptozotocin administered diabetic rats; Pio—pioglitazone-treated STZ rats; Dapa—dapagliflozin-treated STZ rats; DapaPio—dapagliflozin- and pioglitazone-treated STZ rats; *n* = 10–12. Data are presented as mean ± SEM; *p* < 0.05 * vs. control, # vs. STZ, $ vs. Dapa, £ vs. Pio

After 6 weeks of the duration of the experiment, the body weight of STZ rats was significantly lower (*p* < 0.05), while the kidney weight-to-body weight ratio, a marker of DN progression, was significantly higher compared to the control group (*p* < 0.05). Treatment with antidiabetic drugs effectively prevented STZ-induced cachexia. However, only the monotherapies were able to revert the kidney weight-to-body weight ratio to the control values (Table 2). The monotherapies as well as the combined therapy also showed a significant reduction in STZ-induced diabetes symptoms, such as polyuria and increased water intake (*p* < 0.05, Fig. 1).

**Table 2:** Biometric parameters and biomarkers of kidney injury

	Control	STZ	Dapa	Pio	DapaPio
Gravimetry					
Body weight (g)	368 ± 14	267 ± 12*	345 ± 9*#	334 ± 13*#	315 ± 9 *#$£
Left kidney weight (g)	1.21 ± 0.04	1.28 ± 0.09	1.23 ± 0.04	1.23 ± 0.04	1.34 ± 0.03
Left kidney weight/body weight	3.29 ± 0.04	4.38 ± 0.24*	3.77 ± 0.22#	3.77 ± 0.22#	4.28 ± 0.12*£
Biomarkers of kidney injury					
Kim1 (% relative to controls)	100 ± 18	186 ± 25*	109 ± 16#	128 ± 26	121 ± 34#
Nphs1 (% relative to controls)	100 ± 10	63 ± 9*	103 ± 15#	94 ± 15#	77 ± 6*

Groups labelling: *STZ* streptozotocin administered diabetic rats, *Pio* pioglitazone-treated *STZ* rats, Dapa dapagliflozin-treated *STZ* rats, DapaPio dapagliflozin- and pioglitazone-treated*STZ* rats; *n* = 10–12; Kim1 kidney injury molecule, *Nphs1* Nephrin. Data are presented as mean ± *SEM*; *p* < 0.05 * vs. control, # vs. STZ, $ vs. Dapa, £ vs. Pio

### Biomarkers of kidney injury and biochemical parameters

The relative mRNA expression of kidney injury molecule Kim1 in STZ rats was markedly upregulated (vs. control, *p* < 0.05). On the contrary, the expression of Nephrin (Nphs1), an early marker of glomerular injury, was significantly decreased in STZ rats when compared to the non-diabetic controls (*p* < 0.05). At the end of the 6th week, Dapa and DapaPio led to a significant downregulation of Kim1 in the rat kidneys (vs. STZ, *p* < 0.05). Nphs1 gene expression was restored to normal levels by Dapa and Pio monotherapies, but not by the combined DapaPio therapy (Table 2). As shown in Fig. 1, fasting glucose levels, which were elevated in the STZ group, were significantly reduced by both Pio and DapaPio therapies (*p* < 0.05). Diabetes-related albuminuria, indicated by significantly increased 24-h urinary protein excretion, was markedly diminished by all three treatments (vs. STZ, *p* < 0.05). Additionally, STZ rats showed increased serum creatinine and blood urea nitrogen levels, alongside a marked decrease in creatinine clearance. These abnormalities were normalised by the monotherapies as well as the combined therapy (*p* < 0.05, Fig. 1). 

### Blood pressure and heart rate

**Fig. 2:**
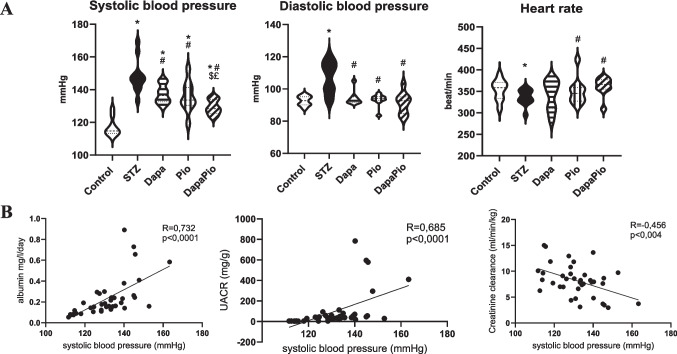
Effect of therapy on blood pressure and heart rate (**A**). Correlation between systolic blood pressure and kidney function parameters: albuminuria, urine albumin-to-creatinine ratio (UACR), creatinine clearance. Groups labelling: STZ—streptozotocin administered diabetic rats; Pio—pioglitazone-treated STZ rats; Dapa—dapagliflozin-treated STZ rats; DapaPio—dapagliflozin- and pioglitazone-treated STZ rats; *n* = 10–12. Data are presented as mean ± SEM; *p* < 0.05 * vs. control, # vs. STZ, $ vs. Dapa, £ vs. Pio

Blood pressure and heart rate assessments showed significantly elevated systolic and diastolic blood pressure in STZ rats, while heart rate was notably lower than in controls (*p* < 0.05). The treatment effectively lowered systolic blood pressure compared to STZ rats, with the combined therapy showing the greatest reduction (*p* < 0.05). Diastolic pressure returned to control levels in all treated groups. Normalisation of heart rate was observed only in the Pio and combined therapy groups (*p* < 0.05, Fig. 2A). Notably, reductions in systolic pressure were positively correlated with kidney function indicators, such as albuminuria and the urine albumin-to-creatinine ratio, while creatinine clearance increased as systolic pressure decreased (*p* < 0.05, Fig. 2B).

### Histology

**Fig. 3:**
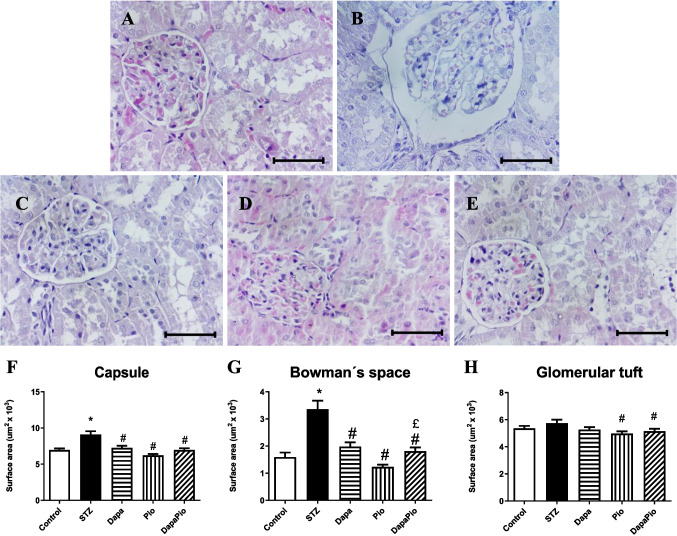
Changes in the glomerular surface area at the histological level. Representative images of glomeruli (hematoxylin and eosin-stained sections) from control (**A**) and diabetic rats (**B**) and rats treated with dapagliflozin (**C**), pioglitazone (**D**) or their combination (**E**). Surface areas of Bowman’s capsule (**F**), Bowman’s space (**G**) and the glomerular tuft (**H**). 400 × magnification; scale bar 50 μm. Groups labelling: STZ—streptozotocin-administered diabetic rats; Pio—pioglitazone-treated STZ rats; Dapa—dapagliflozin-treated STZ rats; DapaPio—dapagliflozin- and pioglitazone-treated STZ rats; *n* = 10–12. Data are presented as mean ± SEM; *p* < 0.05 *vs. control, # vs. STZ, £ vs. PIO

In STZ rats, histological examination revealed pathological changes characteristic of early diabetes such as mesangial expansion, glomerular hypertrophy, light tubular degeneration and glomerulosclerosis. Treatment with Dapa and the combination therapy DapaPio prevented the progression of these changes, as reflected by normal renal glomeruli, minimal degenerative changes in the tubular lining epithelium and mild interstitial fibrosis, indicating an overall protective effect on kidney structure. Pio reduced glomerular hypertrophy but did not completely halt degenerative glomerular and tubular changes (Figs. 3, and 6).

Changes in the surface area of the glomeruli were quantified for all groups as DN results in distinct structural alterations of the kidney. STZ rats showed marked enlargement of the capsule (*p* < 0.05, Fig. 3F) and Bowman’s space (*p* < 0.05, Fig. 3G) when compared with control kidney sections (Fig. 3A). There was only a tendency of glomerular tuft enlargement in the diabetic group, but the difference was not significant (Fig. 3H). The administration of Dapa, Pio and their combination (Fig. 3C–E) significantly reduced the enlargement of Bowman’s capsule (*p* < 0.05, Fig. 3F) and space (*p* < 0.05, Fig. 3G). The surface area of the glomerular tuft was decreased by Pio and its combination with Dapa (*p* < 0.05, Fig. 3H).

### Effect of the therapy on inflammation

#### Expression of proinflammatory markers

**Fig. 4:**
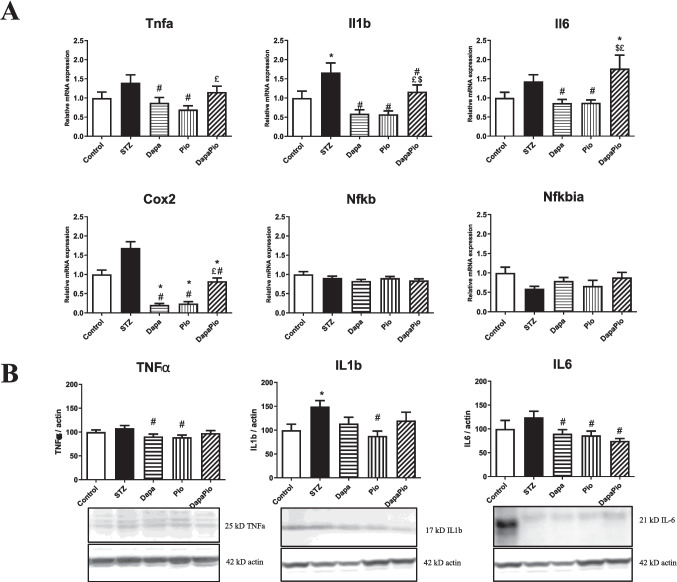
**A** Relative gene expression of proinflammatory markers tumour necrosis factor α (Tnfa), interleukin 1β (Il1b), interleukin 6 (Il6), cyclooxygenase 2 (Cox2), nuclear factor κB (Nfkb) and its inhibitor Nfkbia. **B** Relative expression of proteins involved inflammatory processes TNFα, IL-1β, IL-6. Groups labelling: STZ—streptozotocin administered diabetic rats; Pio—pioglitazone-treated STZ rats; Dapa—dapagliflozin-treated STZ rats; DapaPio—dapagliflozin- and pioglitazone-treated STZ rats; *n* = 10–12. Data are presented as mean ± SEM; *p* < 0.05 * vs. control, # vs. STZ, $ vs. Dapa, £ vs. Pio

STZ-induced hyperglycaemia in rats resulted in a significant increase in Il1b (*p* < 0.05) compared to non-diabetic rats, while only statistically insignificant increases were observed in the mRNA expression of the proinflammatory cytokines Tnfa (tumour necrosis factor α), Il6 (interleukin 6) and the enzyme Cox2 (cyclooxygenase 2). Treatment with Dapa and Pio markedly reduced the gene expression of all four inflammatory markers compared to untreated STZ rats (*p* < 0.05). The combination therapy led to a significant reduction in Il1b and Cox2 gene expression compared to untreated STZ rats (*p* < 0.05); however, the mRNA levels remained substantially increased when compared with monotherapies. The mRNA expression of Nfkb and its inhibitor Nfkbia did not change in any of the experimental groups (Fig. 4A).

Similarly, a significant upregulation in protein levels was observed only in the case of IL1b (vs. control, *p* < 0.05). The protein expression of proinflammatory cytokines TNFα, IL-1β and IL-6 was markedly decreased by the monotherapy (vs. STZ, *p* < 0.05). No additive effect was observed in the group receiving the combined therapy, which only reached the effect of the monotherapies in reducing IL-6 expression (Fig. 4B).

### Immunohistostaining of CD68

**Fig. 5:**
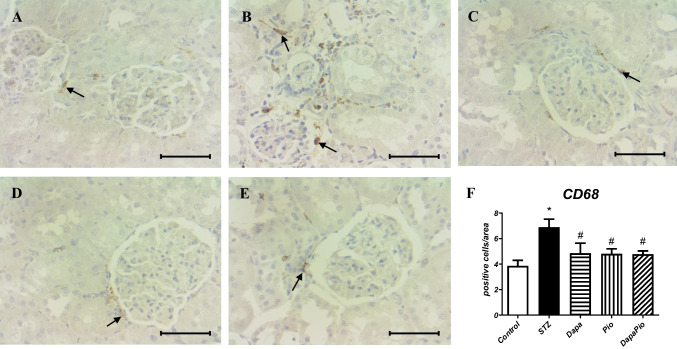
Immunostained renal sections for CD68, a general marker of macrophages, show a normal accumulation of macrophages in the kidneys of control rats (**A**) and a marked increase in accumulation in untreated diabetic rats (**B**). Dapagliflozin (**C**), pioglitazone and their combination (**D**) effectively prevented renal macrophage infiltration. Quantification of macrophage infiltration is expressed as the number of immunopositive cells per defined area (**F**). The arrow indicates CD68-positive regions in kidney sections. (400 × magnification; scale bar 50 μm). Groups labelling: STZ—streptozotocin administered diabetic rats; Pio—pioglitazone-treated STZ rats; Dapa—dapagliflozin-treated STZ rats; DapaPio—dapagliflozin- and pioglitazone-treated STZ rats; *n* = 10–12. Data are presented as mean ± SEM; p < 0.05 *vs. control, # vs. STZ

Immunohistochemical analysis revealed a significantly higher number of cells positively stained for CD68 (glycoprotein expressed in macrophages) in comparison to healthy controls (*p* < 0.05, Fig. 5A, B, F), indicating substantial macrophage infiltration in the kidneys of diabetic rats. The monotherapies and combined therapy effectively reduced this macrophage infiltration induced by diabetic conditions (*p* < 0.05, Fig. 5C–F).

### Effect of the therapy on fibrosis

#### Histological staining (Picro-Mallory trichrome)

**Fig. 6:**
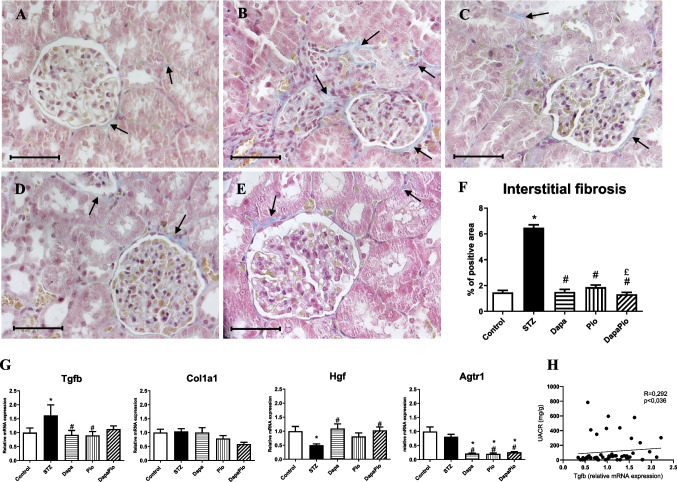
Picro-Mallory-trichrome (PMT) staining of kidney sections. Representative images of glomeruli were obtained from control (**A**) and diabetic rats (**B**) and rats treated with dapagliflozin (**C**), pioglitazone (**D**) or their combination (**E**). Percentage (%) of collagen-positive areas to the total area across four fields in each microscopic section (**F**). Collagen deposition as blue-positive areas indicates fibrotic changes in the tissue (arrow). 400 × magnification; scale bar 50 μm. Relative mRNA expression of genes associated with fibrosis: transforming growth factor-β (Tgfb), collagen type 1, alpha1 (Col1a1), hepatocyte growth factor (Hgf), angiotensin II receptor type 1 (Agtr1) (**G**). Correlation between Tgfb mRNA expression and urine albumin-creatinine ratio (UACR). Groups labelling: STZ—streptozotocin administered diabetic rats; Pio—pioglitazone-treated STZ rats; Dapa—dapagliflozin-treated STZ rats; DapaPio—dapagliflozin- and pioglitazone-treated STZ rats; *n* = 10–12. Data are presented as mean ± SEM; *p* < 0.05 *vs. control, # vs. STZ, £ vs. PIO

Renal tissues of STZ rats stained with Picro-Mallory trichrome showed an obvious increase in collagen deposits (vs. control, *p* < 0.05), indicating extracellular matrix deposition and the presence of tubulointerstitial fibrosis (Fig. 6A, B, F). Markedly reduced collagen-positive areas in rats were observed in all treated groups as compared to the untreated STZ group (*p* < 0.05, Fig. 6C–F).

### Expression of genes associated with fibrosis

The RT-qPCR analysis demonstrated a significant increase of profibrotic cytokine transforming growth factor-β1 (TGF-β1, gene Tgfb) in the STZ rats when compared with that in the control rats, which was significantly attenuated by both Dapa and Pio monotherapy (*p* < 0.05). A similar trend was observed in the group receiving combination therapy; however, it did not reach statistical significance (Fig. 6G). The gene expression of Tgfb was positively correlated with the urinary albumin-creatinine ratio (UACR), indicating its involvement in renal functional deterioration (Fig. 6H). Although experimental diabetes was not associated with increased levels of collagen Col1a1, treatment with Pio and combination treatment led to a mild decrease in Col1a1 expression (Fig. 6G). RT-qPCR analysis also revealed a significant reduction in the protective Hgf (hepatocyte growth factor) expression in diabetic rats, which was restored to control levels by dapagliflozin monotherapy and combination therapy (vs. STZ, *p* < 0.05). The therapy markedly reduced the mRNA levels of angiotensin II receptor type 1 (Agtr1) (vs. control and STZ, *p* < 0.05; Fig. 6H). At a gene level, we did not observe any statistically significant expression changes in the modulation of other profibrotic factors like Col4a3, fibronectin, Smad2 or Smad3 (data not shown).

### Immunohistostaining of α-SMA

**Fig. 7:**
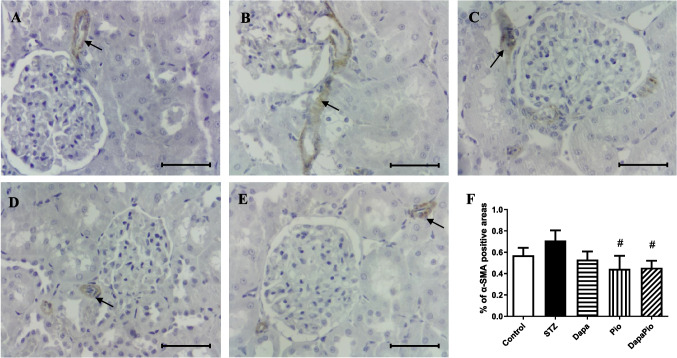
Representative Immunostaining of α-SMA in kidney sections (α-SMA antibody, 400 × magnification; scale bar 50 μm) were obtained from control (**A**) and diabetic rats (**B**) and rats treated with dapagliflozin (**C**), pioglitazone (**D**) or their combination (**E**). Percentage (%) of α-SMA-positive areas to total area across four fields in each microscopic section (**F**). The arrow indicates α-SMA-positive regions in kidney sections. Groups labelling: STZ—streptozotocin administered diabetic rats; Pio—pioglitazone-treated STZ rats; Dapa—dapagliflozin-treated STZ rats; DapaPio—dapagliflozin- and pioglitazone-treated STZ rats; *n* = 10–12. Data are presented as mean ± SEM; *p* < 0.05 # vs. STZ

Kidney sections of control rats examined by immunohistochemical analysis showed almost undetectable α-SMA (alpha-smooth muscle actin)-immunopositive areas in glomerular, tubular and microcapillary compartments. In the STZ group, we observed their increased occurrence in the evaluated areas; however, this increase was not statistically significant compared to the control group (Fig. 7A, B, F). Treatment with Pio, but not Dapa, revealed a pronounced decline in renal α-SMA-positive areas compared to untreated STZ rats (*p* < 0.05, Fig. 7C, D, F). Albeit Dapa failed to attenuate α-SMA expression in the kidneys of STZ rats, the combination with Pio produced a significant reduction in α-SMA expression versus untreated ones (Fig. 7E, F).

## Discussion

Current treatment strategies may slow, but often fail to stop, the progression toward ESRD. To develop effective therapies for preventing and slowing DN, gaining new strategies and insights into its pathophysiology is mandatory (Kanasaki et al. 2013). In this study, we used an STZ-induced diabetic rat model to assess the combined effects of the SGLT2 inhibitor dapagliflozin and PPARγ agonist pioglitazone in the kidneys. Despite improvements in renal function markers (creatinine clearance, albuminuria, UACR, BUN, urine output), the combination therapy did not offer significant renoprotective advantages over monotherapy, except for its superior reduction of systolic blood pressure. Importantly, the combined therapy as well as the monotherapies effectively slowed down the progression of renal dysfunction and pathological processes leading to kidney injury and morphological changes. Both drugs reduced the production of proinflammatory cytokines together with macrophage infiltration, while also regulating the expression of fibrosis-related factors and collagen deposition. α-SMA positive areas were downregulated exclusively by pioglitazone and the combined therapy. However, overall, the combination therapy did not enhance renoprotection beyond what was achieved with monotherapy.

Several experimental studies have shown that STZ-treated rats begin to exhibit early signs of DN, including albuminuria, hyperfiltration, elevated blood urea nitrogen, reduced creatinine clearance and structural changes in the kidneys within 4 to 6 weeks, consistent with our findings (Luippold et al. 2016; Oraby et al. 2019; Feng et al. 2021). STZ administration also induced classic diabetic symptoms, such as hyperglycaemia, polydipsia, polyuria and body weight reduction. Dapagliflozin effectively prevented T1DM-related cachexia, despite its association with increased leptin levels, osmotic diuresis and body weight reduction (Kralova et al. 2021). Pioglitazone produced a similar effect, likely due to its side effect of fluid retention, which may have contributed to body weight preservation (Yang and Soodvilai 2008). Notably, the combined therapy also significantly prevented body weight loss, although the final body weight was lower than in the monotherapy groups. This may be attributed to sodium excretion induced by dapagliflozin, which could counterbalance the fluid retention effect of pioglitazone (Han et al. 2018). Urine output data in our study support this hypothesis, indicating reduced fluid burden on the cardiovascular system. The marked increase in urine output with combined dapagliflozin and pioglitazone may also reflect additive effects on renal function. Dapagliflozin promotes glucosuria and osmotic diuresis, while pioglitazone may enhance sodium and water excretion via improved insulin sensitivity and vascular function. Consequently, the combination potentiates diuretic effects, consistent with previous reports showing that SGLT2 inhibitors with insulin-sensitising agents amplify renal fluid excretion without harming renal function (Lo et al. 2023). Additionally, the therapy significantly reduced both systolic and diastolic blood pressure, with the most pronounced effect observed in the combined therapy group. Additionally, the therapy significantly reduced both systolic and diastolic blood pressure, with the most pronounced effect observed in the combined therapy group. Notably, both monotherapy and combination therapy also reduced mean arterial pressure, as recently reported, highlighting an additional beneficial effect (Cinakova et al. 2023). Hypertension is a critical factor in the progression of DN and simultaneously an indicator of declining renal function. Indeed, in our study, blood pressure reduction by both monotherapy and combined therapy was associated with the prevention of morphological changes and significant improvement in renal function markers, such as albuminuria, creatinine clearance and their ratio (UACR) with minimal effect on blood glucose levels. Furthermore, the recently published effects of this therapy on glomerulosclerosis further support and strengthen our current positive observations (Cinakova et al. 2025). These findings align with those of Masoad et al., who demonstrated a strong relationship between blood pressure reduction and improved renal function after pioglitazone administration (Masoad et al. 2012). Conversely, data from Tanimoto et al. showed an improvement in the glomerular-to-Bowman’s capsule volume ratio and UACR without significant changes in fasting glucose levels or systemic blood pressure in the early stages of experimental DN (Tanimoto et al. 2004). Improvement in renal function following dapagliflozin administration in our study is consistent with recent clinical studies in patients with chronic kidney disease (CKD), with and without type 2 diabetes (Jongs et al. 2021). While the combined therapy produced the most pronounced decrease in systolic blood pressure, it did not yield an additive effect on renal outcomes. The aforementioned findings suggest a renoprotective effect of both drugs, driven by mechanisms beyond their glucose-lowering actions. However, our observations also imply the presence of pharmacodynamic or compensatory mechanisms that may limit a further enhancement in renoprotection with combined therapy.

Kidney injury molecule-1 (KIM-1) is an early biomarker for tubular damage, undetectable in healthy renal systems (Khan et al. 2019). Its elevated expression correlates strongly with kidney damage severity and is linked to inflammatory and fibrotic processes in the kidney tubular system (Humphreys et al. 2013; Song et al. 2019). In our study, the most pronounced increase in KIM-1 expression was observed in the untreated diabetic group, while significant reductions were noted with dapagliflozin monotherapy and, similarly, with combined therapy. This decline in KIM-1 expression may be also attributed to restored redox balance and mitigated hypoxia (Cinakova et al. 2023; Chen et al. 2023). Interestingly, pioglitazone monotherapy did not significantly reduce KIM-1 expression, consistent with our previous findings, where pioglitazone failed to fully protect against kidney damage (Cinakova et al. 2025). In contrast, a study by Medić et al. demonstrated pioglitazone’s dose-dependent ability to lower KIM-1 levels in a model of gentamicin-induced nephrotoxicity, highlighting its potential role as a protective agent when combined with nephrotoxic drugs (Medić et al. 2019). Conversely, the expression of nephrin, another early marker of DN, was positively modulated exclusively by the monotherapies, suggesting the restoration of the renal glomerular filtration barrier. This occurred despite minimal effects on glycaemic levels, even though hyperglycaemia is known to directly destabilise nephrin, leading to increased urinary protein leakage (Tung et al. 2018; Kostovska et al. 2020). Notably, albuminuria was also improved with the combined therapy, highlighting the involvement of multiple mechanisms contributing to enhanced renal function.

We hypothesised that the functional and histopathological changes in diabetic kidneys were driven by inflammation and the propagation of fibrotic processes. Supporting this, the kidneys of diabetic rats in our study showed significantly elevated gene and protein expression of several pro-inflammatory cytokines, along with a marked increase in macrophage infiltration. Chronic inflammation stimulates immune cell activation, which in turn activates intrinsic renal cells, promoting the production and release of profibrotic cytokines and growth factors. This cascade ultimately leads to renal fibrosis, the hallmark of progressive DN (Imig and Ryan 2013)*.* Both pioglitazone and dapagliflozin decreased proinflammatory markers expression (IL1b, IL6, Cox2, Tnfα) and CD68-positive areas. Minor discrepancies observed between gene and protein expression levels may result from standard post-translational modifications (Wang 2008). These findings align with previous studies highlighting the anti-inflammatory properties of dapagliflozin and pioglitazone (Oraby et al. 2019; Abdollahi et al. 2022; El Gazzar et al. 2023). The anti-inflammatory effect of dapagliflozin may stem from its ability to inhibit TLR-4 overexpression, leading to reduced macrophage polarisation regardless of glucose concentrations (Abdollahi et al. 2022; Cai et al. 2023). Additionally, dapagliflozin downregulates NF-κB, a central transcription factor that regulates the production of key inflammatory cytokines, as demonstrated in non-diabetic cardiorenal disease model (Urbanek et al. 2023). However, no changes in NF-κB or its inhibitor were detected across any experimental group in our study. Similarly, pioglitazone has been shown to reduce pro-inflammatory cytokine expression via agonism on PPARγ receptors in monocytes and lymphocytes in individuals with impaired glucose tolerance (Ko et al. 2008; Zhang et al. 2008). It achieves these effects also without affecting glucose levels by targeting the PPARγ/miRNA‑124/STAT3 signalling pathway (El Gazzar et al. 2023). Interestingly, combination therapy showed a somewhat attenuated effect on cytokine gene expression, suggesting a potential plateau or interaction effect at the subcellular level without significant pharmacokinetic interactions (Kasichayanula et al. 2011).

Inflammatory cells and cytokines are key drivers of fibroblast activation, leading to the destruction of normal kidney architecture and functional decline (Kanasaki et al. 2013). In our study, the therapy alleviated fibrosis and partially or fully reduced α-SMA positive areas to the control values probably via targeting TGF-β signalling pathway. Our findings align with prior studies demonstrating the antifibrotic effects of dapagliflozin in experimental T1DM, where α-SMA downregulation correlated with reduced collagen deposition (Oraby et al. 2019). Dapagliflozin-mediated downregulation of TGF-β reduces extracellular matrix (ECM) accumulation, thereby inhibiting extensive fibrotic remodelling in tubulointerstitial and glomerular regions (Urbanek et al. 2023). This reduction in TGF-β further corroborates dapagliflozin’s antifibrotic properties, as TGF-β is widely recognised as a central driver of DN-related fibrosis (Zhuang et al. 2019). Through TGF-β signalling, fibrogenic processes disrupt the filtration barrier by thickening the glomerular basement membrane and depleting nephrin, leading to proteinuria and initiating epithelial-to-mesenchymal transition in the renal tubular epithelium (Ghayur and Margetts 2013). Additionally, fibrosis modulation may be supported by restored levels of HGF, which counteracts TGF-β signalling and inhibits the initiation and progression of chronic renal fibrosis (Liu 2004). In our study, Hgf expression was elevated by dapagliflozin and the combined therapy, further supporting their antifibrotic potential. Elevated angiotensin II levels further enhance TGF-β activation, upregulating other fibrogenic molecules. Notably, our therapy significantly reduced angiotensin II receptor type 1 (Agtr1) expression, potentially contributing to lower vascular resistance and ECM accumulation within the mesangium and tubulointerstitium (Burns 2000). Similar to dapagliflozin, pioglitazone has demonstrated robust antifibrotic effects. Toblli et al. reported that pioglitazone downregulates at subtherapeutic doses fibronectin and connective tissue growth factor, and at higher doses, it effectively prevents TGF-β-induced renal fibrosis by inhibiting EGR-1 and STAT3, while counteracting additional profibrotic processes such as autophagy dysfunction and miRNA dysregulation (Toblli et al. 2011; Németh et al. 2019; Manzéger et al. 2023). Depending on dosage and combination, pioglitazone may exert even greater effects in downregulating profibrotic markers (Amano et al. 2018). Collectively, these findings confirm that both dapagliflozin and pioglitazone mitigate DN progression in STZ rats by suppressing tubulointerstitial fibrosis and glomerulosclerosis. Histological and molecular biology analyses suggest that their antifibrotic actions are mediated through distinct pathways and molecular targets, offering complementary therapeutic mechanisms.

This study has several limitations. First, the method of drug administration via chow, rather than gavage, introduced slight variability; however, overall consistency was maintained within each experimental group. Second, although we used standard doses and acknowledged the dose-dependent nature of these drugs, adjusting the doses—potentially lowering them—could modify the outcomes by reducing drug interactions and minimising the risk of adverse effects (Toblli et al. 2011). Finally, the duration of experimental diabetes (6 weeks) may limit the findings, as a longer period would likely exacerbate structural and functional renal changes (Oraby et al. 2019). However, extending the duration posed ethical concerns due to increased mortality risks in the untreated diabetic group, and our primary objective was to evaluate the drugs’ effects on early-stage DN.

## Conclusion

In summary, this study demonstrates that while both dapagliflozin and pioglitazone individually exert glucose control-independent renoprotective effects in a streptozotocin-induced model of diabetic nephropathy, their combination does not confer additional advantages. Both agents independently reduced markers of inflammation and fibrosis, as evidenced by decreased proinflammatory cytokine levels and macrophage infiltration, alongside modulation of profibrotic factors, leading to improved renal morphology and function. Interestingly, while the combined treatment demonstrated an enhanced antihypertensive effect, it did not surpass the renoprotective benefits beyond those achieved with monotherapy. These findings suggest that while SGLT2 inhibition and PPARγ activation each offer direct renal protective effects beyond glucose lowering, their concurrent application provides limited additive benefits for managing inflammation and fibrosis in experimental T1DM-related diabetic nephropathy. 

## Supplementary Information

Below is the link to the electronic supplementary material.

ESM 1 (PDF 423 KB)

## Data Availability

The datasets used and/or analysed during the current study are available from the corresponding author upon reasonable request.
