# Supplementary material for: Combined dapagliflozin and pioglitazone therapy in diabetic nephropathy: no added benefit beyond monotherapy in inflammation and fibrosis
Source: Naunyn Schmiedebergs Arch Pharmacol. 2025 Sep 26;399(2):3001–15. doi: 10.1007/s00210-025-04613-x (PMC12901098; doi:10.1007/s00210-025-04613-x)
Supplement: Supplementary file 1 — (PDF 423 KB) [file 210_2025_4613_MOESM1_ESM.pdf]

Combined Dapagliflozin and Pioglitazone Therapy in Diabetic Nephropathy: No Added Benefit Beyond Monotherapy in inflammation and fibrosis

Naunyn-Schmiedeberg's Archives of Pharmacology

Cinakova A, Vavrincova-Yaghi D, Vavrinec P, Krenek P, Klimas J, Kralova E.*

Comenius University Bratislava, Faculty of Pharmacy, Department of Pharmacology and Toxicology,

SK-832 32 Bratislava, Slovakia

*Corresponding Author: Eva Kralova

Address: Comenius University Bratislava, Faculty of Pharmacy, Department of Pharmacology and Toxicology, Kalinciakova 8, SK-832 32 Bratislava, Slovakia

Tel: +421 25 01 17 363

Email: kralova5@uniba.sk

ORCID: 0009-0000-8993-9011

full unedited gel for Figure 4B (TNFα)

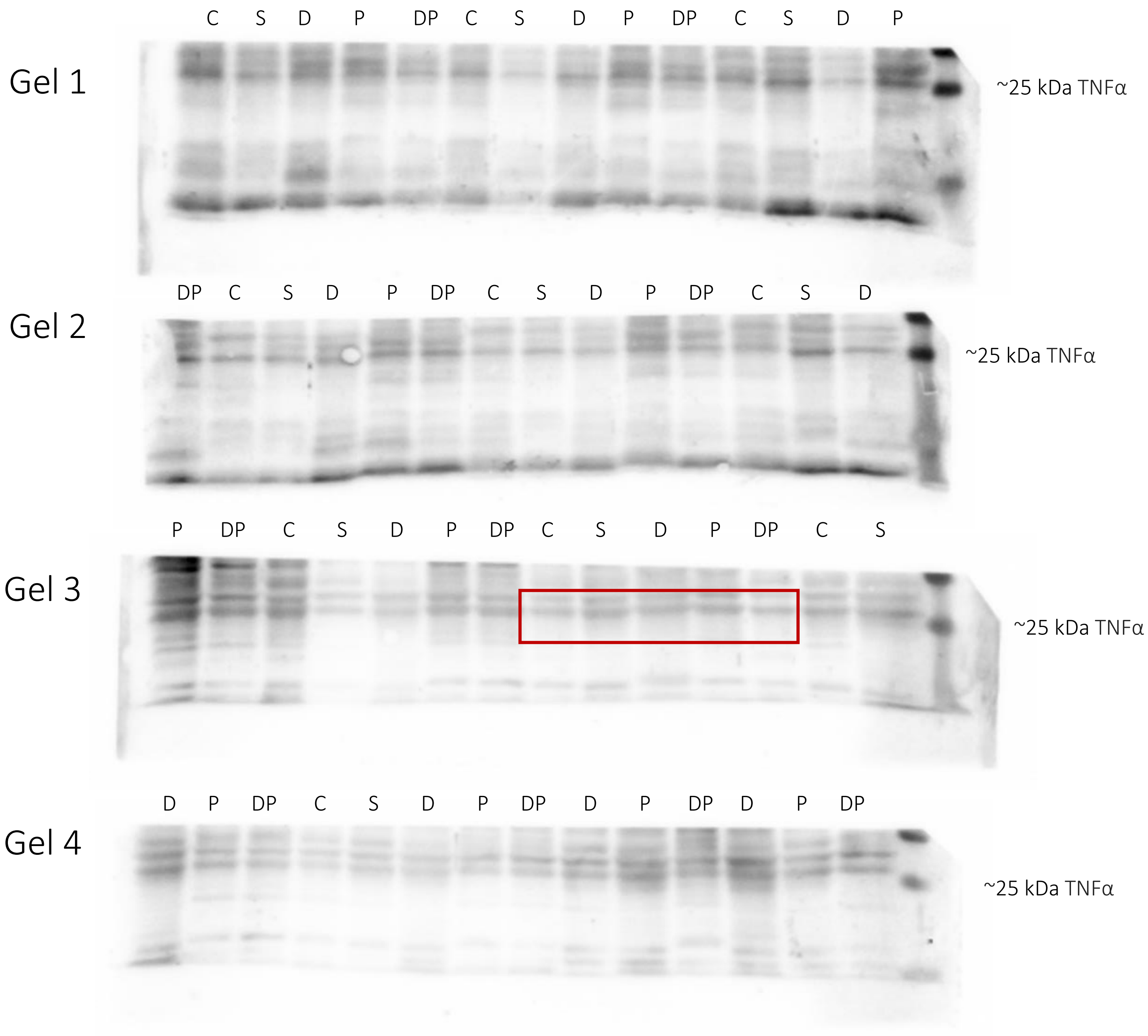

C= Control (10)
S= STZ (10)
D= Dapagliflozin (12)
P= Piogliazone (12)
DP= DapaPio (12)

full unedited gel for Figure 4B (IL1b)

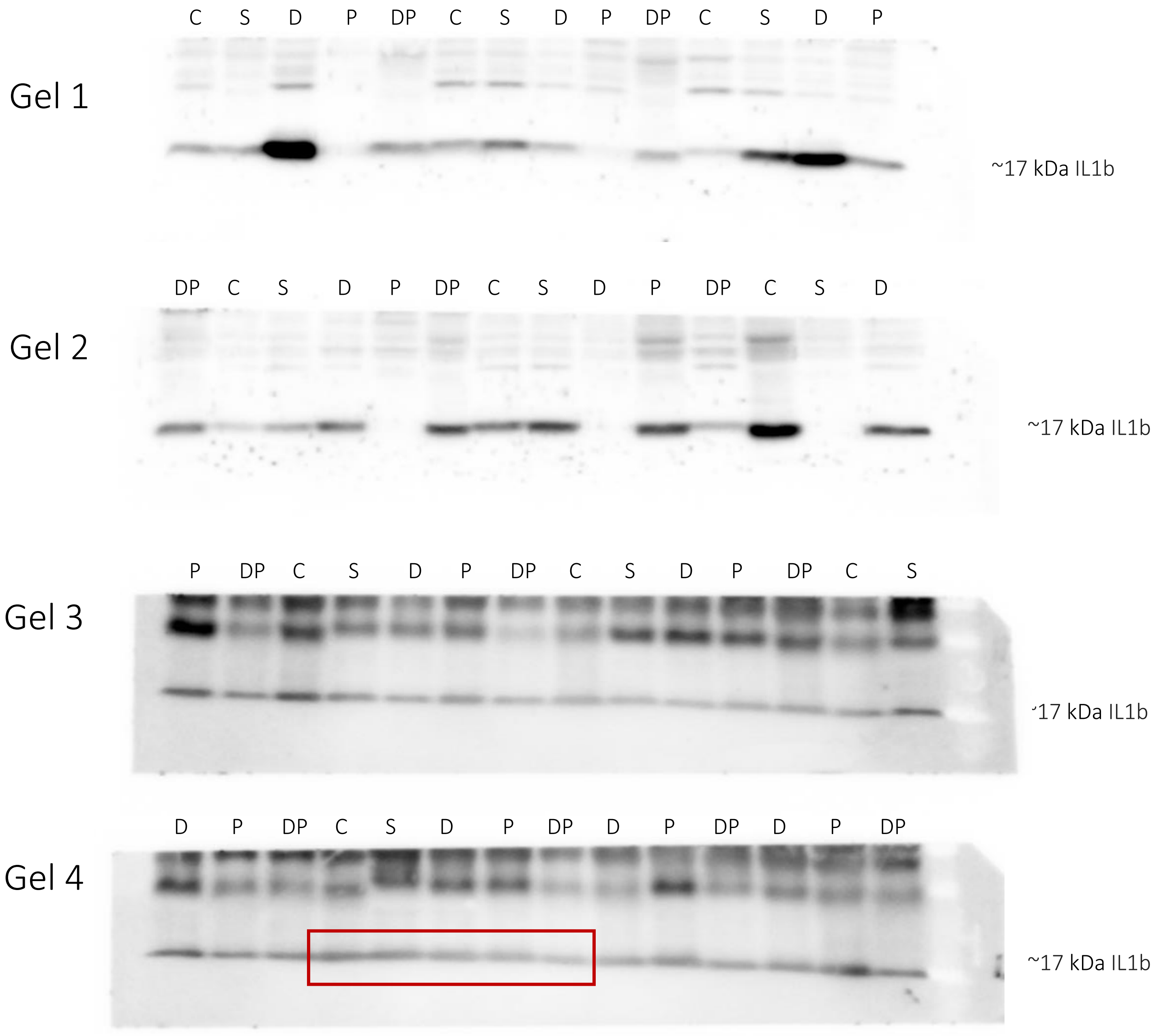

C= Control (10)
S= STZ (10)
D= Dapagliflozin (12)
P= Piogliazone (12)
DP= DapaPio (12)

full unedited gel for Figure 4B (IL6)

Gel 1

C S D P DP C S D P DP C S D P

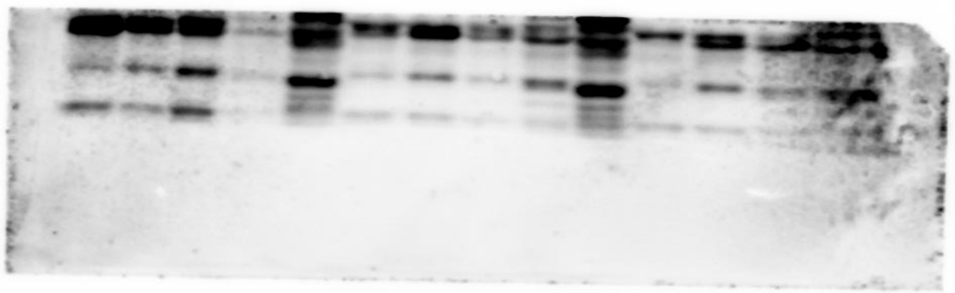

~21 kDa IL6

Gel 2

DP C S D P DP C S D P DP C S D

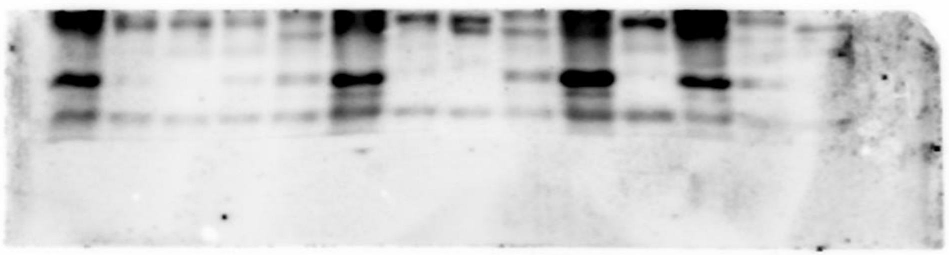

~21 kDa IL6

Gel 3

P DP C S D P DP C S D P DP C S

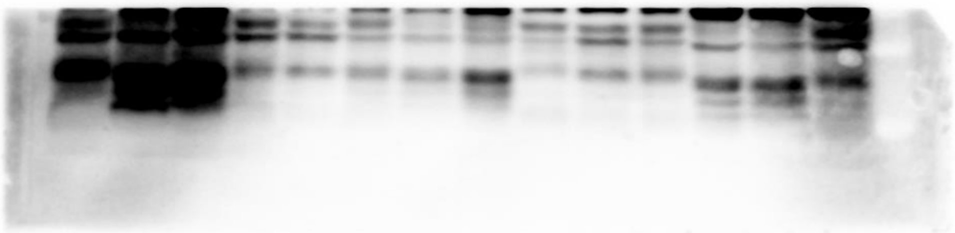

~21 kDa IL6

Gel 4

D P DP C S D P DP D P DP D P DP

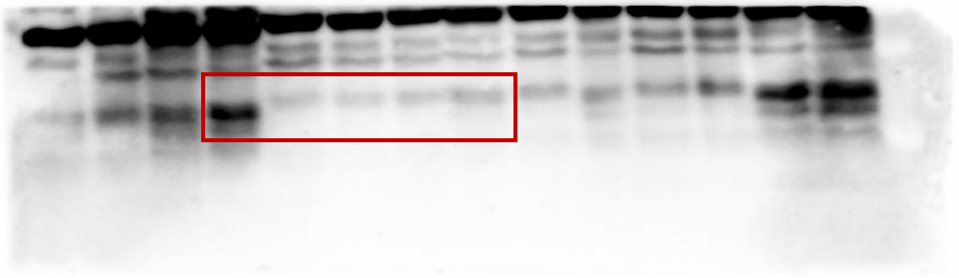

~21 kDa IL6

C= Control (10)
S= STZ (10)
D= Dapagliflozin (12)
P= Piogliazone (12)
DP= DapaPio (12)

Full unedited gel for housekeeping protein actin beta (~42kDa)
-normalisation of the following proteins:
IL1b, IL6 (Pages 3,4)

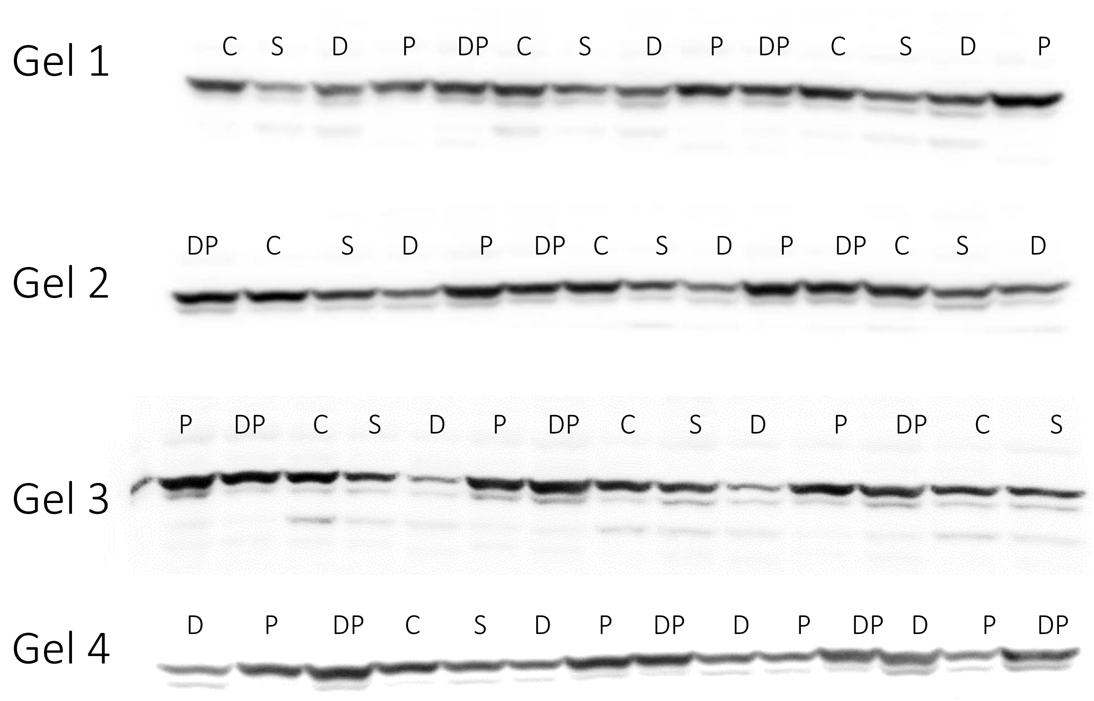

Full unedited gel for housekeeping protein actin beta (~42kDa)
-normalisation of the following proteins:
TNFα (Page 2)

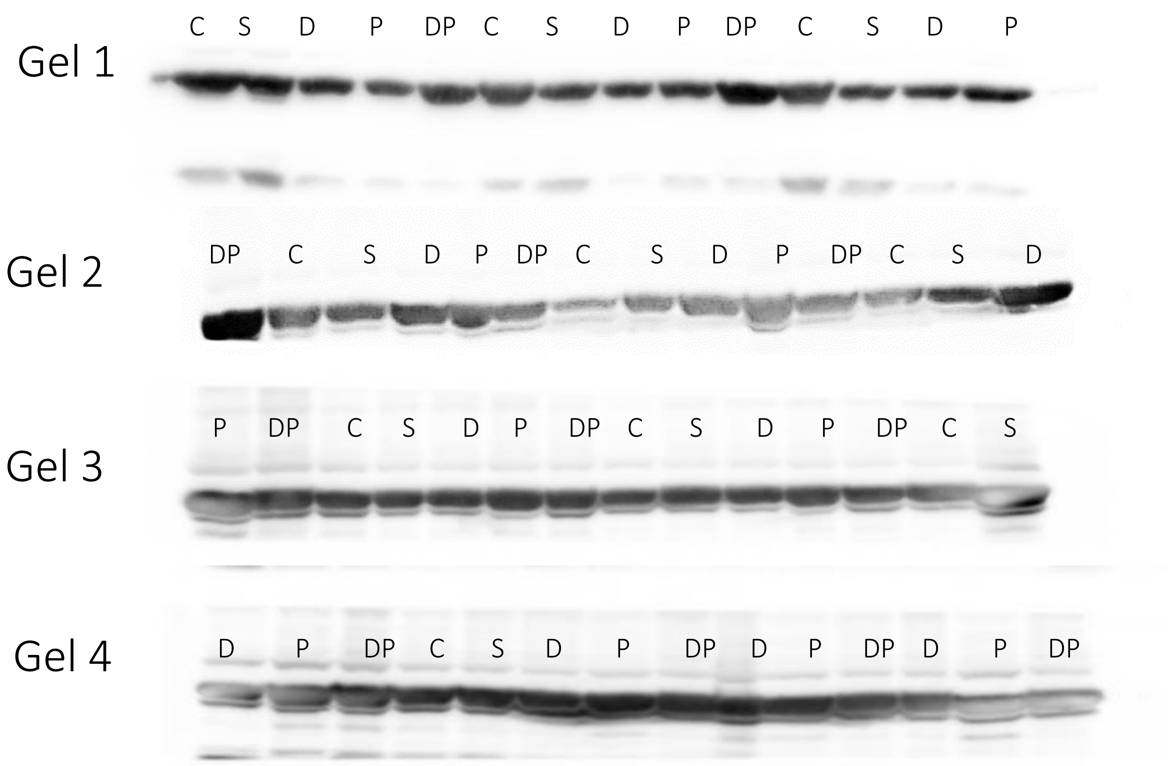

Representative immunoblot regions for the reference protein actin were created by cropping the relevant area corresponding to the examined protein.
